# Influence of marginal incision and Le Fort I osteotomy on periodontal tissues: a prospective longitudinal study

**DOI:** 10.1007/s10266-022-00721-9

**Published:** 2022-06-23

**Authors:** Lauren Bohner, Rômulo Maciel Lustosa, Thomas Stamm, Marcel Hanisch, Johannes Kleinheinz, Susanne Jung

**Affiliations:** 1 Department of Cranio-Maxillofacial Surgery, University Hospital Muenster, Albert-Schweitzer-Campus 1 Gebäude W30, 48149 Muenster, Germany; 2 Department of Periodontology, State University of Maringá, Maringá, A. Colombo 5790, 87020-900 Brazil; 3 Department of Orthodontics, University Hospital Muenster, Albert-Schweitzer-Campus 1 Gebäude W30, 48149 Muenster, Germany

**Keywords:** Orthognathic surgical procedure, Jaw surgery, Dental papillae, Periodontium

## Abstract

The purpose of this prospective study was to evaluate whether the surgical protocol involving marginal incision and Le Fort I osteotomy affects the periodontal outcome. Twenty-nine patients requiring surgical correction of maxillary dysgnathia were selected for this study. Periodontal conditions of maxillary anterior teeth were assessed prior to the surgery (*T*_0_), one month (*T*_1_), and 6 months (*T*_2_) after the surgical procedure. Interdental papillae loss, periodontal parameters and aesthetic outcomes in the anterior zone were assessed. Statistical analysis was performed with Friedman´s test and within ANOVA (*p* ≤ 0.05) followed by post-hoc tests. Papilla height decreased from *T*_0_ to *T*_1_ (*p* = 0.003), followed by an increase from *T*_1_ to *T*_2_ (*p* = 0.040). PPD (*T*_0_ = 1.72 ± 0.46; *T*_2_ = 2.13 ± 0.43) and CAL (*T*_0_ = 1.24 ± 0.55; *T*_2_ = 1.99 ± 0.70) increased after 6 months. There were statistically significant differences in aesthetic score among *T*_0_, *T*_1_ and *T*_2_. Periodontal changes after orthognathic surgery were within acceptable biological levels. Although a change in papillae height was reported after the surgery, initial values were recovered after 6 months. Surgical incision design might improve the outcome of Le-Fort I osteotomy. Anterior aesthetic zone may benefit from a marginal incision design, as it respects vascularization principle and may ensure a wound healing without complications.

**Supplementary Information:**

The online version contains supplementary material available at 10.1007/s10266-022-00721-9.

## Introduction

Le Fort I osteotomy is a well-established technique to correct maxillary discrepancies, as open bite, anterior vertical excess of maxilla or transverse maxilla deficiency [1, 2]. When properly planned, the technique shows minimal intra and postoperative complications, being highly indicated for correction of maxillary position [3]. Despite the advantages of the technique, concerns regarding its risks still exist due to the proximity between important anatomical structures and osteotomized sites [4]. A recent systematic review stated that from 2078 patients who underwent a Le Fort I osteotomy, 177 suffered from any intrasurgical or postoperative complication [3].

In this regard, damage of periodontal tissue can lead to esthetic and biological injuries [5]. Nonetheless, the occurrence of periodontal trauma after orthognathic surgery is still controversial [2, 6–8]. Carroll et al. [7] founded no alteration of periodontal parameters after orthognathic surgery in 40 patients [7]. Conversely, an assessment performed in a follow-up of 4–10 years revealed several periodontal lesions in almost 50% of the evaluated osteotomy sites [8].

Periodontal tissue changes may be related to the compromised blood flow resulting from soft tissue incision and osteotomies. According to previous studies, reduced gingival blood flow may damage soft tissue [9] and lead to bone loss, scar formation and delayed wound healing [10].

In order to avoid complications, surgical incision must respect and preserve the natural anatomy of soft tissue [10]. Traditional Cupar method consists of a labial approach using a circumvestibular incision, which is related with a low complication rate [11–13]. However, alternative incision designs have also been proposed. Because of the lack of studies available in literature, it is still unknown whether different surgical incisions affect periodontal health.

In this context, Kleinheinz et al. [10] suggested that a marginal incision design could benefit anterior aesthetic zone by respecting vascularization principle and ensuring wound healing without complications [10]. However, although this technique has been widely used for periodontal and oral surgeries [14], few studies report its use during orthognathic surgery. Thus, the benefits of a marginal incision design are still unknown. For this reason, the purpose of this study was to assess both clinical and aesthetic outcomes of periodontal tissues after a surgical protocol involving marginal incision and Le-Fort I osteotomy.

## Materials and methods

### Trial design

This prospective longitudinal study was conducted at the Department of Oral and Maxillofacial Surgery, University Hospital Münster, after approval from the Ethics Committee of the University of Münster (2017-609-f-S). Study´s protocol was described according to the STROBE Guidelines [15]. Informed consent was obtained from all subjects involved in the study.

Sample size was calculated based on a previous study, and considering papillae height as a primary outcome [16]. To assess a mean difference of 0.3 mm ± 0.4 mm (*α*  = 0.05, power of 95%), 26 patients were required. Considering a possible withdrawal of approximately 10%, 29 patients were selected for this study.

Periodontal outcomes of patients requiring surgical correction of maxillary dysgnathia were assessed in three different periods: a day prior to each surgery (*T*_0_), one month (*T*_1_) and 6 months (*T*_2_) after surgery.

### Eligibility criteria

As inclusion criteria, patient must show optimal periodontal health, good oral hygiene and absence of dental implants, crowns or restorations in the measurement site. Exclusion criteria comprised patients under 18 years of age, pregnant patients or patients presenting some systemic disease that could compromise healing after surgery.

### Outcome

The primary outcome was the height of interproximal papillae at anterior maxillary teeth (Canine–canine). Secondary outcomes consisted of periodontal parameters (probing depth, bleeding on probing, Plaque index, clinical attachment level, gingival recession) and esthetic outcomes.

### Surgical procedure

All cases were planned according the “Digital model surgery system” of University Hospital Münster [17] and operated by the same surgeon (S.J.). Orthognathic surgery was conducted under general anesthesia with nasal endotracheal intubation. A marginal incision with bilateral relaxing incision was performed from the second premolar site to the contralateral tooth. In order to preserve blood supply, a palatal incision was avoided. After raising the flap, a bilaterally osteotomy was performed above the teeth apex. Anterior nasal septum was separated from the maxilla with a U-shaped osteotome. Next, flap was raised bilaterally at tuberosity, and the pterygomaxillary disjunction was performed with a chisel. After down-fracture and mobilization of the maxilla, palatal bone was osteotomized from one premolar site to the contralateral site. In addition, if required, bone was removed to allow maxillary impaction. Osteotomized maxillary segments were repositioned and aligned into the desired position using miniplates, maxillomandibular fixation and occlusal splint. After, the flap was repositioned and a running-suturing was performed with polyglactin 910 (Vicryl 3-0, Vicryl, Ethicon).

### Post-operative treatment

Intermaxillary fixation was performed with ligature wires during the first day after surgery followed by the use of elastic power chain for 3 days. Orthodontic treatment continued until a satisfactory occlusion was achieved. Post-operative care included the use of chlorhexidine 0.2% for oral irrigations instead of teeth brushing. Special diet included feeding tube in the first three days, followed by liquid diet for the next three days and soft diet during 10 days.

### Measurements

Maxillary jaws were scanned in different times (*T*_0_, *T*_1_ and *T*_2_) with an intra-oral scanner (Trios, 3Shape), providing an accurate imaging of soft tissues [18]. Papillae height was determined as the distance (mm) of a line connecting the zenith of adjacent teeth to the papillae tip. Measurements were performed by a blinded examiner using the software Geomagic Verify (3D Systems). After positioning the model according to the three-dimensional plane determined by the software, a line connecting the zenith of anterior teeth was drawn. Single points marking the papillae tip were determined, and the distance between this line to each interproximal point was automatically calculated using a measurement tool.

Periodontal parameters were assessed by bleeding on probing (BoP), plaque index (PI), probing pocket depth (PPD), clinical attachment level (CAL) and gingival recession (GR). Measurements were taken clinically by two calibrated examiners using a periodontal probe. Calibration was conducted on a pilot study (*n* = 3), on which each examiner probed on the same patient six sites of each tooth. Scores of bleeding on probing and plaque index were assessed according to a classification proposed on a previous study [19]. A mean rate was calculated by dividing the total score by the number of examined teeth. BoP, PPD and CAL were measured at six sites around the tooth (mesiolabial, labial, distolabial, mesiolingual, lingual, and distolingual). PI and GR were measured only in the vestibular surface of anterior maxillary teeth.

Additionally, esthetic outcome was determined by a modified version of Pink Esthetic Score (mPES) [20]. Mesial papillae, distal papillae and level of soft-tissue margin at anterior zone (canine-canine) were assessed by two blinded examiners (L.B., M.H.). Each point was assessed with a score rate from 0 to 2, being 0 the worst and 2 the best outcome [16].

### Statistical analysis

Statistical analysis was conducted using the software SPSS 26 (IBM Corp.) and considering a significance level of *p* < 0.05. Adherence to normal curve was assessed with Shapiro–Wilk’s test. ANOVA for repeated measurements and Friedman’s test were used to assess to assess periodontal parameters at each period (*T*0, *T*1, *T*2). Bonferroni-adjusted post-hoc analysis was used for statistical significant differences.

Kappa’s test was used to assess inter-examiner reliability for periodontal calibration and esthetic outcome. Reliability was categorized according Altman et al. [21], as: weak (≤ 0.20), reasonable (0.21–0.40), moderate (0.41–0.60), good (0.61–0.80) very good (0.81–1.00). Correlation between papilla height and periodontal parameters was analyzed with Pearson´s and Spearman´s correlation tests.

## Results

A total of 17 female and 12 male patients with a mean age (± standard deviation) of 25.82 (± 10.38 years) participated in this study. In general, participants presented good health conditions, and only one of them had a previous disease (Asperger’s syndrome).

As part of the treatment, all patients have been previously treated with fixed orthodontic appliances. In addition, two patients underwent a surgical maxillary expansion two years prior to the orthognathic surgery. For all patients, a bimaxillary orthognathic surgery with one-piece Le-Fort I osteotomy was conducted. Individual patient´s features and surgical planning details are described in Online Appendix 1.

### Primary outcome

**Fig. 1:**
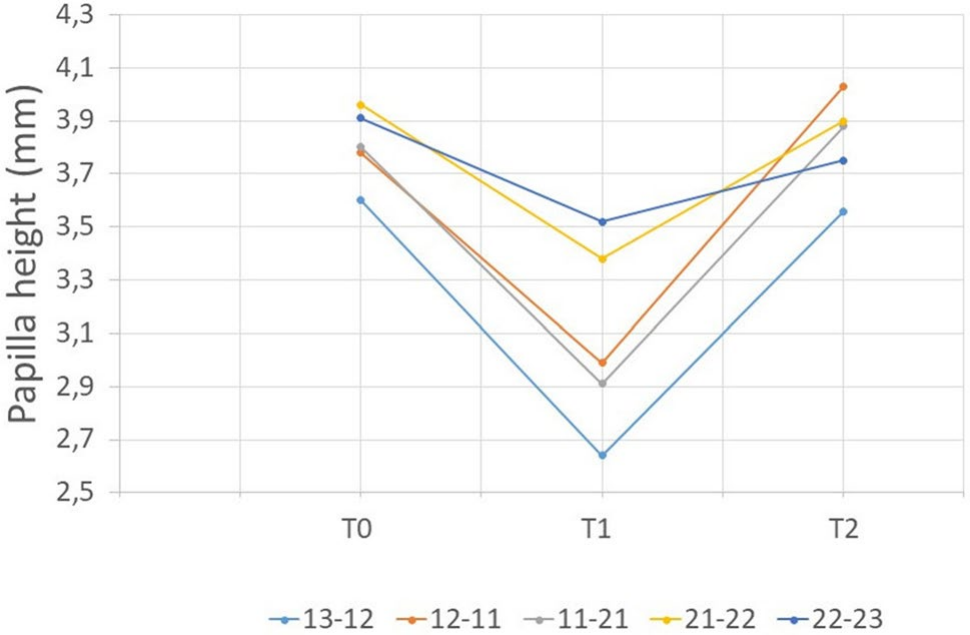
The graphic represents the mean values of papilla height (mm) prior to the surgical procedure (*T*_0_), 1 month (*T*_1_) and 6 months (*T*_2_) postoperatively. Papillae height values tend to decrease at *T*_1_ and increase at *T*_2_

Papilla height decreased significantly from *T*_0_ to *T*_1_ (*p* = 0.003) and increased from *T*_1_ to *T*_2_ (*p* = 0.040), recovering in T2 mean values close to the one showed on the baseline (*p* > 0.05), as shown in Table 1 and Fig. 1.

**Table 1:** Mean values of papilla height (mm) prior to the surgical procedure (*T*_*0*_), 1 month (*T*_1_) and 6 months (*T*_2_) postoperatively

Measurement site	Papillae height (mm)			ANOVA (*p* value)	Post-hoc analysis (*p* value)		
	*T*_0_	*T*_1_	*T*_2_		*T*_0_ − *T*_1_	*T*_0_ − *T*_2_	*T*_1_ − *T*_2_
13–12	3.60 ± 0.86	2.64 ± 1.06	3.56 ± 1.02	0.011*	0.016*	1.000	0.126
12–11	3.78 ± 0.58	2.99 ± 0.78	4.03 ± 0.78	0.001*	0.021*	1.000	0.005*
11–21	3.80 ± 0.87	2.91 ± 1.40	3.88 ± 1.03	0.025*	0.102	1.000	0.113
21–22	3.96 ± 0.45	3.38 ± 0.50	3.90 ± 0.57	0.013*	0.021	1.000	0.168
22–23	3.91 ± 0.89	3.52 ± 0.79	3.75 ± 0.72	0.248	0.439	1.000	1.000
Mean values	3.81 ± 0.59	3.09 ± 0.76	3.82 ± 0.58	0.003*	0.020*	1.000	0.040*

^*^Means statistically significant difference (*p* < 0.05)

### Secondary outcomes

With regard to periodontal measurements, examiners showed a high concordance for periodontal probing. There was a slight increase in PPD and CAL between T0 and T2, which was not considered clinically significant [22]. There was no statistical significant difference among treatments with respect to gingival recession, PI and BoP (Table 2). Papilla height showed a positive correlation between gingival recession (Rs = 0.699, *p* = 0.008) and CAL (Rp = 0.545, *p* = 0.05), and was negatively correlated with BoP (Rs = − 0.546, *p* = 0.05).

**Table 2:** ANOVA comparison for T0, T1, T2

Periodontal parameters	*T*_0_	*T*_1_	*T*_2_	ANOVA (*p* value)	Multiple comparison (*p* value)		
					*T*_0_-*T*_1_	*T*_0_-*T*_2_	*T*_1_-*T*_2_
PPD (mm)	1.72 ± 0.46	1.79 ± 0.51	2.13 ± 0.43	0.007*	1.000	0.022*	0.084
GR (mm)	0.20 ± 0.33	0.14 ± 0.25	0.16 ± 0.25	0.432			
CAL (mm)	1.24 ± 0.55	1.37 ± 0.51	1.99 ± 0.70	< 0.001*	1.000	0.002*	0.001*
PI	0.13 ± 0.26	0.06 ± 0.15	0.19 ± 0.37	0.076			
BoP	0.08 ± 0.09	0.05 ± 0.09	0.17 ± 0.20	0.109			

*PPD* probing pocket depth, *GR* gingival recession, *CAL* clinical attachment level, *PI* plaque index, *BoP* bleeding on probing

^*^Means statistical significant difference

**Fig. 2:**
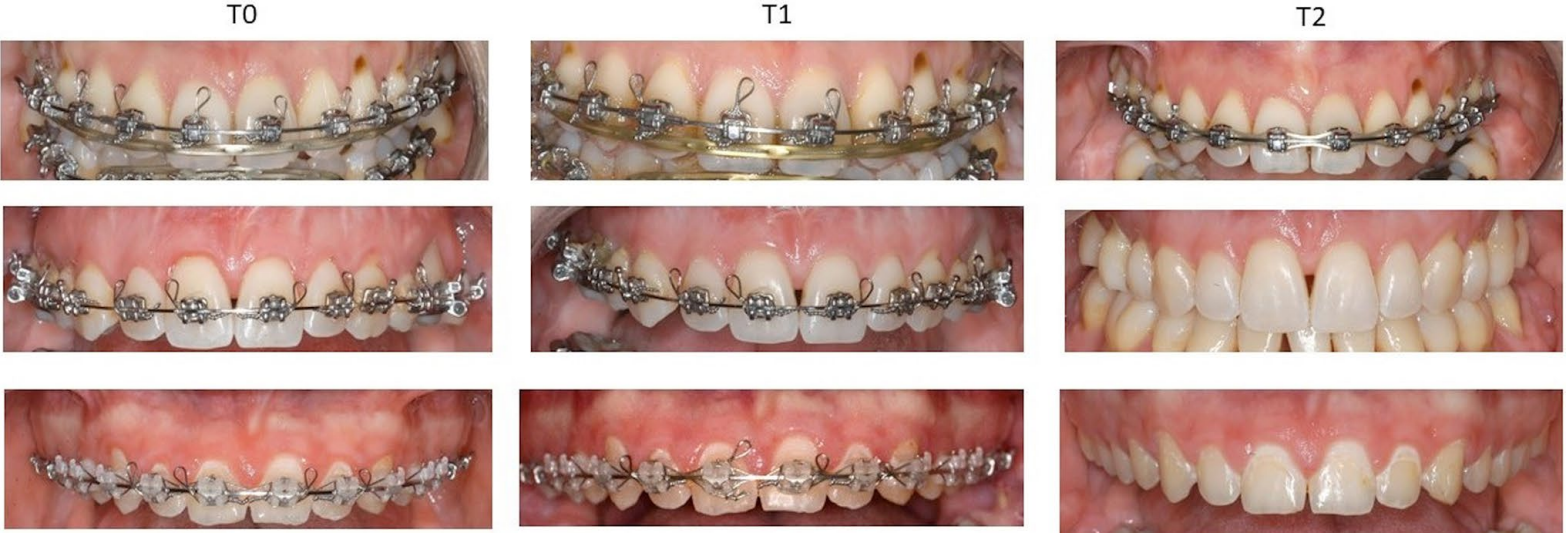
Comparison among gingival tissue of three patients at *T*_0_, *T*_1_ and *T*_2_. Observe the stabilization of mucosa appearance and absence of scar tissues

Esthetic analysis showed no statistically significant difference (*p* = 0.652) among mPES values at *T*_0_ (5.65 ± 0.54), *T*_1_ (5.66 ± 0.51) and *T*_2_ (5.38 ± 0.84). A good or very good concordance between investigators was observed in T0 (0.923), *T*_1_ (0.874) and T_2_ (0.800). Scar-like tissues were not observed in clinical assessment of oral mucosa (Fig. 2).

## Discussion

The findings of this study suggested that a marginal incision followed by a Le-Fort I osteotomy does not result in significant periodontal changes 6 months after orthognathic surgery. Even though a papillae height loss was observed one month later, it seemed to be recovered after the healing process. Furthermore, although probing pocket depth increased over time, it remained limited to a physiological level and did not impair the biological tissues [23]. In this respect, periodontal changes were considered as temporary and did not affect the anterior teeth.

Pathological alterations in periodontal tissues observed during the first month after surgery might be related to postoperative conditions. Pain, swelling and reduced mouth opening, as well as intermaxillary fixation with splint, can make difficult oral hygiene and lead to increased plaque accumulation [24]. Weinspach et al. [23] showed a modification of periodontal flora in the first six weeks after surgery [23]. However, in this study, plaque accumulation did not differ significantly among the observation periods.

In contrast to our findings, previous reports showed a significant increase of periodontal pockets and gingival recession 6 weeks after surgery [5, 7, 23]. Studies showed a negative relationship between periodontal modifications and interdental osteotomy from the canine to the second premolar [2, 7, 8], suggesting that bone dehiscence can be a predisposing factor for periodontal changes [23]. However, these changes were restricted to the interdental osteotomy site, and no periodontal alterations were reported at anterior maxilla.

Morphological alterations in periodontal tissue are related with reduction in gingival blood flow after surgical incision and osteotomy [9, 24, 25]. Since alveolar vessels are transected, palatal pedicle becomes the main source of blood supply, which decreases in 50% one hour following surgery [9]. Ischemic zones may result in avascular necrosis, leading to sequelae, as periodontal defects or teeth injuries [9, 26]. In this regard, surgical incision may optimize the wound healing, provided that natural blood vascularization and soft tissue anatomy are respected [10].

The surgical protocol adopted in this study is related with minimal periodontal alterations 6 months after surgery. Likewise, the authors suggested that a marginal incision may be suitable for maintaining long-term stability of periodontal tissues. This become increasingly significant under certain circumstances in which soft tissue management is required, i.e., dental implant in patients with tooth agenesis.

However, a marginal incision relies up on the gingival phenotype and can be technically challenging. Thus, a thinner mucosa constitutes an obstacle in performing the technique and is accompanied by lower vascularization, which may result on bone loss and gingival recession. In these cases, a conventional circumvestibular incision may be preferable to avoid periodontal complications [10, 14, 26, 27]. Since this study did not evaluate different incision designs, comparison of surgical techniques should be considered in future studies.

Osteotomy may also play an important role in reducing blood flow. A recent study showed that the percentage rate of post-operative complications after Le-Fort I osteotomy was significantly higher for segmental osteotomies in comparison to one-piece Le-Fort I osteotomy [28]. Sancar et al. [11] associated these post-operative complications to the compromised blood flow, which can be affected by segmental osteotomies. The authors suggest that one-piece Le-Fort I osteotomy may be more suitable for preventing periodontal complications [11]. In this study, a one-piece Le-Fort I osteotomy was performed to conduct all operations, and no conclusion can be reached regarding the osteotomy type.

Esthetic analysis showed no significant difference among the evaluated periods. Scars prevention and preservation of papillae architecture ensured the satisfactory esthetic outcomes related to this surgical protocol. Nonetheless, undesired esthetic outcomes of periodontal tissues after an orthognathic surgery are not reported in literature, suggesting that pink esthetic is preserved regardless of the adopted surgical protocol.

The present study has some limitations. Since patients underwent a second surgical procedure after a period of 6–9 months for plates removal, further periodontal changes are expected. Furthermore, patient’s features, skeletal asymmetry and treatment plan may influence biological and esthetic outcomes. In addition, oral hygiene, presence of anterior tooth-supported prosthesis, and gingival phenotype may affect the periodontal tissue after an orthognathic surgery [29]. Since patient´s individual variations were not considered in the analysis, these results should be interpreted with caution. The findings of this study resulted from a complex treatment planning and should not be completely attributed to the chosen surgery protocol.

## Conclusions

Papillae height loss was reversible and recovered values similar to the baseline after 6 months. A surgical protocol involving marginal incision and Le-Fort I osteotomy did not result in clinically significant changes in periodontal tissues and did not influence esthetics at anterior zone.

## Supplementary Information

Below is the link to the electronic supplementary material.

Supplementary file1 (DOCX 15 KB)
