# Supplementary material for: Influence of marginal incision and Le Fort I osteotomy on periodontal tissues: a prospective longitudinal study
Source: Odontology. 2022 Jun 23;111(1):201–6. doi: 10.1007/s10266-022-00721-9 (PMC9810686; doi:10.1007/s10266-022-00721-9)
Supplement: Supplementary file 1 — Supplementary file1 (DOCX 15 KB) [file 10266_2022_721_MOESM1_ESM.docx]

**Table 1.** Description of patient´s features and surgical procedures.

| **Patients** | Age | Diagnosis | | Therapy | Maxillary osteotomy |
| --- | --- | --- | --- | --- | --- |
|  |  | Maxilla | Mandible |  |  |
| **1** | 27 | Maxillary retrognathism | Mandibular rethrognathism | Bimaxillary orthognathic surgery | Le-Fort-I Osteotomy |
| **2** | 24 | Maxillary retrognathism | Mandibular rethrognathism | Bimaxillary orthognathic surgery | Le-Fort-I Osteotomy, previous surgical maxillary expansion |
| **3** | 27 | Maxillary retrognathism | Mandibular rethrognathism | Bimaxillary orthognathic surgery | Le-Fort-I Osteotomy |
| **4** | 22 | Maxillary retrognathism | -- | Bimaxillary orthognathic surgery | Le-Fort-I Osteotomy |
| **5** | 23 | Maxillary retrognathism | Mandibular prognathism | Bimaxillary orthognathic surgery | Le-Fort-I Osteotomy |
| **6** | 22 | Maxillary retrognathism | Mandibular prognathism | Bimaxillary orthognathic surgery | Le-Fort-I Osteotomy |
| **7** | 26 | Maxillary retrognathism | Mandibular prognathism | Bimaxillary orthognathic surgery | Le-Fort-I Osteotomy |
| **8** | 20 | Maxillary retrognathism, open bite | Mandibular prognathism | Bimaxillary orthognathic surgery | Le-Fort-I Osteotomy |
| **9** | 27 | Maxillary retrognathism | -- | Bimaxillary orthognathic surgery | Le-Fort-I Osteotomy |
| **10** | 22 | Maxillary retrognathism | Mandibular prognathism | Bimaxillary orthognathic surgery | Le-Fort-I Osteotomy |
| **11** | 42 | Deep overbite | Mandibular rethrognathism | Bimaxillary orthognathic surgery | Le-Fort-I Osteotomy |
| **12** | 20 | Open bite | Mandibular rethrognathism | Bimaxillary orthognathic surgery | Le-Fort-I Osteotomy, previous surgical maxillary expansion |
| **13** | 27 | Gummysmile | Mandibular rethrognathism | Bimaxillary orthognathic surgery | Le-Fort-I Osteotomy |
| **14** | 32 | Maxillary retrognathism | Mandibular prognathism | Bimaxillary orthognathic surgery | Le-Fort-I Osteotomy |
| **15** | 37 | Maxillary retrognathism | Mandibular rethrognathism | Bimaxillary orthognathic surgery | Le-Fort-I Osteotomy |
| **16** | 24 | -- | Mandibular prognathism | Bimaxillary orthognathic surgery | Le-Fort-I Osteotomy |
| **17** | 32 | Maxillary retrognathism | Mandibular prognathism | Bimaxillary orthognathic surgery | Le-Fort-I Osteotomy |
| **18** | 25 | Maxillary retrognathism | Mandibular prognathism | Bimaxillary orthognathic surgery | Le-Fort-I Osteotomy |
| **19** | 20 | Maxillary retrognathism | Mandibular rethrognathism | Bimaxillary orthognathic surgery | Le-Fort-I Osteotomy |
| **20** | 20 | Maxillary retrognathism | Mandibular prognathism | Bimaxillary orthognathic surgery | Le-Fort-I Osteotomy |
| **21** | 22 | Maxillary retrognathism | Mandibular rethrognathism | Bimaxillary orthognathic surgery | Le-Fort-I Osteotomy |
| **22** | 20 | Maxillary retrognathism | Mandibular prognathism | Bimaxillary orthognathic surgery | Le-Fort-I Osteotomy |
| **23** | 20 | Maxillary retrognathism | Mandibular prognathism | Bimaxillary orthognathic surgery | Le-Fort-I Osteotomy |
| **24** | 18 | Maxillary retrognathism | Mandibular prognathism | Bimaxillary orthognathic surgery | Le-Fort-I Osteotomy |
| **25** | 24 | - | Mandibular prognathism | Bimaxillary orthognathic surgery | Le-Fort-I Osteotomy |
| **26** | 34 | Maxillary retrognathism | Mandibular prognathism | Bimaxillary orthognathic surgery | Le-Fort-I Osteotomy |
| **27** | 18 | Maxillary retrognathism | - | Bimaxillary orthognathic surgery | Le-Fort-I Osteotomy |
| **28** | 20 | Maxillary retrognathism | Mandibular prognathism | Bimaxillary orthognathic surgery | Le-Fort-I Osteotomy |
| **29** | 27 | Maxillary retrognathism | Mandibular prognathism | Bimaxillary orthognathic surgery | Le-Fort-I Osteotomy |
